# Fourier Transform Infrared Spectroscopy Application for *Candida auris* Outbreak Typing in a Referral Intensive Care Unit: Phylogenetic Analysis and Clustering Cut-Off Definition

**DOI:** 10.3390/microorganisms12071312

**Published:** 2024-06-27

**Authors:** Antonio Curtoni, Lisa Pastrone, Miriam Cordovana, Alessandro Bondi, Giorgia Piccinini, Mattia Genco, Paolo Bottino, Carlotta Polizzi, Lorenza Cavallo, Narcisa Mandras, Silvia Corcione, Giorgia Montrucchio, Luca Brazzi, Cristina Costa

**Affiliations:** 1 Department of Public Health and Paediatrics, University of Turin, 10126 Turin, Italy; antonio.curtoni@unito.it (A.C.); giorgia.piccinini@unito.it (G.P.); mattia.genco@unito.it (M.G.); paolo.bottino@unito.it (P.B.); cpolizzi@cittadellasalute.to.it (C.P.); lorenza.cavallo@unito.it (L.C.); narcisa.mandras@unito.it (N.M.); cristina.costa@unito.it (C.C.); 2 Microbiology and Virology Unit, Department of Laboratory Medicine, University Hospital Città della Salute e della Scienza di Torino, 10126 Turin, Italy; 3 Bruker Daltonics GmbH & Co. KG, 28359 Bremen, Germany; 4 PhD National Programme in One Health Approaches to Infectious Diseases and Life Science Research, Department of Public Health, Experimental and Forensic Medicine, University of Pavia, 27100 Pavia, Italy; 5 Infectious Diseases, Department of Medical Sciences, University of Turin, 10126 Turin, Italy; silvia.corcione@unito.it; 6 School of Medicine, Tufts University, Boston, MA 02111, USA; 7 Department of Surgical Sciences, University of Turin, 10126 Turin, Italy; giorgiagiuseppina.montrucchio@unito.it (G.M.); luca.brazzi@unito.it (L.B.); 8 Intensive Care and Emergency, Department of Anaesthesia, University Hospital Città della Salute e della Scienza di Torino, 10126 Turin, Italy

**Keywords:** *Candida auris*, Fourier transform infrared spectroscopy, IR-biotyper, outbreak, phylogenetic analysis, cluster analysis, multi-resistant fungal pathogen, healthcare-associated infections

## Abstract

Recently *Candida auris* has emerged as a multi-resistant fungal pathogen, with a significant clinical impact, and is able to persist for a long time on human skin and hospital environments. It is a critical issue on the WHO fungal priority list and therefore it is fundamental to reinforce hospital surveillance protocols to limit nosocomial outbreaks. The purpose of this study was to apply Fourier transform infrared spectroscopy (FT-IR) to investigate the phylogenetic relationships among isolated strains from a *C. auris* outbreak at the University Intensive Care Unit of a Tertiary University hospital in Turin (Italy). To calculate a clustering cut-off, intra- and inter-isolate, distance values were analysed. The data showed the presence of a major Alfa cluster and a minor Beta cluster with a defined *C. auris* clustering cut-off. The results were validated by an external *C. auris* strain and Principal Component and Linear Discriminant Analyses. The application of FT-IR technology allowed to obtain important information about the phylogenetic relationships between the analysed strains, defining for the first time a “not WGS-based” clustering cut-off with a statistical–mathematical approach. FT-IR could represent a valid alternative to molecular methods for the rapid and cost-saving typing of *C. auris* strains with important clinical implications.

## 1. Introduction

*Candida auris* was first isolated from the external ear canal of a Japanese patient in 2009 [1]. Since then, it has been responsible for several infections worldwide with a serious nosocomial health risk [2]. In fact, this yeast merged pivotal pathogenetic features to pose a threat to public healthcare systems such as antifungal multidrug resistance, biofilm formation, ability to spread through contact transmission, and opportunistic behaviour in critically ill and immunocompromised patients [3]. Furthermore, reliable identification by traditional phenotypic and biochemical methods is challenging as *C. auris* can be misidentified as other yeasts (e.g., *Candida haemulonii*, *Candida lusitaniae*, *Candida guilliermondii*) and its correct identification at the species level requires more advanced techniques such as DNA sequencing, Nucleic Acid Amplification Tests, or, most frequently, Matrix-Assisted Laser Desorption Ionization Time-of-Flight Mass Spectrometry (MALDI-TOF MS) [4,5].

After its identification as a novel species, *C. auris* emerged in Africa, India (2009), China (2011), and South (2012) and North America (2013) [6]. In the same year, *C. auris* reached Europe with sporadic cases in the United Kingdom [7]. In the period 2013–2021, 1812 new cases were reported by 15 countries belonging to the European Union and European Economic Area (EU/EEA). For most cases, carriage status was reported (1146; 63.2%), while a bloodstream or another type of infection accounted for 277 (15.3%) and 186 (10.3%) patients, respectively [8]. Focusing on Italy, *C. auris* was first identified as being responsible for invasive infection in a hospital in the Liguria region in 2019 [9]. Thence, other 360 cases were registered until December 2022 in four Northern Italy regions (Liguria, 297; Piedmont, 48; Emilia-Romagna, 15; Veneto, 1) [10]. During this time span, the *C. auris* epidemic raised two peaks as follows: the first occurred in December 2020 and was related to Liguria local epidemiology, while the second was observed in September 2022, mainly because of the emergence of an outbreak in a referral Intensive Care Unit (ICU) in Turin (Piedmont) [10,11].

With regard to *C. auris* infection-control strategies, international healthcare agencies and expert consensus recommend disinfection of high-touch areas and reusable equipment twice or three times daily in rooms housing patients infected by *C. auris*. Chlorine-based agents or other sporicidal disinfectants belonging to List P of the Environmental Protection Agency (EPA) Registered Disinfectants are highly effective in controlling *C. auris* cross-transmission and its spread in healthcare settings [12,13]. Moreover, contact tracing of *C. auris*-positive patients is critical to prevent yeast diffusion and to determine potential outbreaks. IR-Biotyper (IR-BT) (Bruker Daltonics GmbH & Co. KG, Bremen, Germany), a novel typing instrument based on Fourier transform infrared (FT-IR) spectroscopy, is able to define a biochemical fingerprint of bacteria, mycobacteria, and yeast, which is useful to compare and determine if there is a correlation among different microorganisms. Considering this, IR-BT represents a promising tool for rapid and reliable microbial outbreak analysis in nosocomial settings [14,15,16].

A preliminary analysis of the *C. auris* outbreak that occurred between July 2021 and March 2022 in the A.O.U. Città della Salute e della Scienza, Turin, Italy, was performed by Corcione *et al*. in 2022, which considered the epidemiological and clinical aspects [11].

The aim of this work was to broaden and deepen the previous outbreak analysis by the application of IR-BT in order to investigate the phylogenetic relationship among *C. auris* strains isolated from a referral ICU of A.O.U. Città della Salute e della Scienza of Turin, a Tertiary University hospital in Piedmont, Italy [11]. For this purpose, for the first time, data collected from IR-BT analysis were used to experimentally define a clustering cut-off to classify new *C. auris* strains as belonging to the same cluster or not.

## 2. Materials and Methods

This study was performed in the Microbiology and Virology Unit of the University Hospital A.O.U. Città della Salute e della Scienza of Turin, Italy, between December 2021 and February 2023. Positive clinical and surveillance samples for *C. auris* belonging to patients admitted to the university ICU were considered in the present work. Only the first strain per patient was considered. Subsequent to the diagnostic routine, the isolated *C. auris* strains were rendered irreversibly anonymous. The anonymization process was carried out in accordance with the procedure concerning the use of clinical data for scientific purposes and of biological samples stored in laboratories for retrospective studies. Successively, the strains were stored at −80 °C.

### 2.1. C. auris Surveillance Protocol

**Figure 1:**
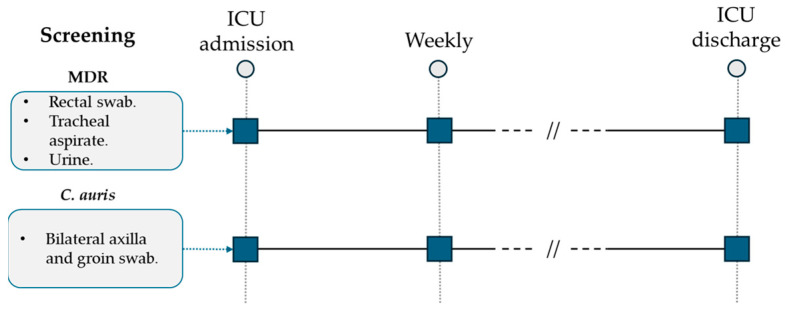
ICU multidrug-resistant bacteria and *C. auris* surveillance protocol.

All patients admitted to the ICU were screened weekly by standard surveillance cultures (urine culture, endotracheal aspirate, rectal swab) for multidrug-resistant (MDR) bacteria detection (Figure 1). Following the first evidence of a *C. auris*-positive urine culture during surveillance standard screening and according to international available guidelines, a specific surveillance protocol was developed and applied for all patients hospitalized in the ICU ward [17,18,19,20,21]. *C. auris* screening was performed weekly through a bilateral axilla and groin eSwab (Copan, Brescia, Italy) before patient hygiene nursing care. After sample collection, they were seeded on BD Sabouraud Agar with gentamicin and chloramphenicol (Becton Dickinson, Franklin Lakes, NJ, USA) and on chromogenic agar (Brilliance Candida Agar, Thermo Scientific, Waltham, MA, USA). Incubation was performed at 37 °C for 48 h. All the isolated yeasts were identified to the species level and reported using Microflex LT MALDI-TOF (Bruker Daltonics GmbH & Co. KG, Bremen, Germany) according to the manufacturer’s instructions.

### 2.2. IR-Biotyper Protocol for C. auris Typing

Stored *C. auris* strains were thawed and grown at 37 °C on BD Sabouraud Agar for 24 h and then subcultivated on the same medium for 24 + 1 h for IR-BT measurements. Sample preparation was performed according to the manufacturer’s instructions. Briefly, two highly filled 1 µL inoculation loops of yeast colonies were suspended in 50 μL of 70% ethanol solution in suspension vials containing metal beads included in the IR-BT kit (Bruker Daltonics GmbH & Co. KG). The suspension was homogenized by vortexing for two minutes, and then 50 μL of deionized water was added to reach the final volume (100 μL). After two minutes of vortexing, 10 μL of the yeast suspension was spotted in five technical replicates on a silicon sample plate (Bruker Daltonics GmbH & Co. KG) and dried at room temperature. Two IR test standards (Bruker Daltonics GmbH & Co. KG) were included in duplicate in each run. All samples were tested in triplicate (biological replicates) on three different days to overcome the biological variation present in cultural and IR-BT tests.

Spectra were acquired and analysed in transmission mode (spectral range: 4000–500 cm^−1^) through OPUS v.8.2 and IR Biotyper Client v.4.0 software (Bruker Daltonics GmbH & Co. KG). The second-order derivative was calculated using the Savitzky–Golay algorithm on nine datapoints, and spectra were vector-normalized. The similarity analysis among the spectra was carried out with a hierarchical clustering algorithm based on Euclidean single linkage. The above-mentioned software allowed us to construct a distance matrix and a dendrogram, which gives a tree-like overview of the spectral relationship. Moreover, the unsupervised dimensionality reduction method, Principal Component Analysis (PCA), and supervised Linear Discriminant Analysis (LDA), which maximizes the inter-group variance and minimizes the intra-group variance, were performed to represent the spectra as dots in the two-dimensional (2D) and three-dimensional (3D) scatter plots. Each strain was represented with an average spectrum derived from fifteen IR-BT measurements (five spectra, technical replicates, for three biological replicates).

In order to interpret the local *C. auris* outbreak correctly, a highly characterized strain, DSM 21092, belonging to DSMZ-German Collection of Microorganisms and Cell Cultures GmbH (Leibniz Institute, Braunschweig, Germany) was included as external clustering control.

### 2.3. Statistical Approach to Define a Clustering Cut-Off for IR-Biotyper and C. auris

Starting from the distance matrix, intra-strain variation (span between technical and biological replicates of the same isolate) and inter-strain variation (span between technical and biological replicates of different isolates) were evaluated for each *C. auris* strain.

In order to define a *C. auris* clustering cut-off, intra- and inter-strain values, average, median, standard deviation, 5th percentile, and 95th percentile were calculated and graphed. A 5% error was considered acceptable for clustering discrimination.

If the inter-isolate distance between two strains was included within the 95th percentile of the intra-isolate distances, the first strain was considered similar to the technical and biological replicates of the others; therefore, it could be defined as clonal. Assuming this, the 95th percentile of intra-isolate distances was considered the lower cut-off to define a strain as part of the same cluster (minimum clustering cut-off). On the other hand, if the inter-isolate distance between two strains was under the 5th percentile of the inter-isolate distance between two distinct groups, the strains could be considered part of the same cluster. Therefore, the 5th percentile of inter-isolates distances between two clusters was considered as the higher cut-off to consider a strain as belonging to a different cluster (maximum clustering cut-off). The area between the lower and higher cut-off values was defined as a grey zone of uncertain clustering results.

## 3. Results

During the considered period of 14 months, 56 positive cases of *C. auris* were identified in patients admitted to the ICU. The majority of *C. auris*-positive samples (51/56; 91.1%) resulted from surveillance cultures from patients with colonization at the skin level (45; 80.4%) and the urinary (three; 5.4%) and respiratory tract (three; 5.4%) levels. In five cases (8.9%), an invasive infection occurred, with *C. auris* isolation from blood cultures and intravascular catheter tip samples (Table 1).

A total of 855 spectra was obtained by typing the 56 clinical strains of *C. auris* involved in the outbreak and the external control strain. Intra- and inter-isolate distances of the acquired spectra were analysed in order to obtain a reference clustering cut-off.

### Analysis of Intra-Isolate and Inter-Isolates Distance Values and Clustering Cut-Off Definition

**Figure 2:**
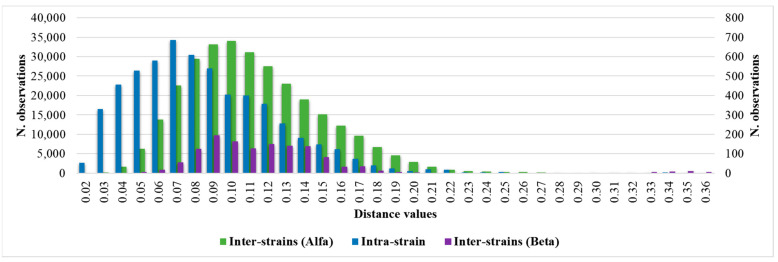
Intra-strain and Alfa and Beta inter-strains distance values distributions.

Considering the intra-isolate distances of each strain of *C. auris* under study, the mean, median, minimum, maximum, standard deviation, 5th percentile, and 95th percentile were calculated. The histogram of the frequencies of all intra-isolate distance values showed a Gaussian distribution with a unimodal trend (Figure 2). The median and the 5th and 95th percentiles were 0.080, 0.030, and 0.160, respectively. The intra-isolate distance value analysis is reported in detail in the Supplementary Materials.

**Figure 3:**
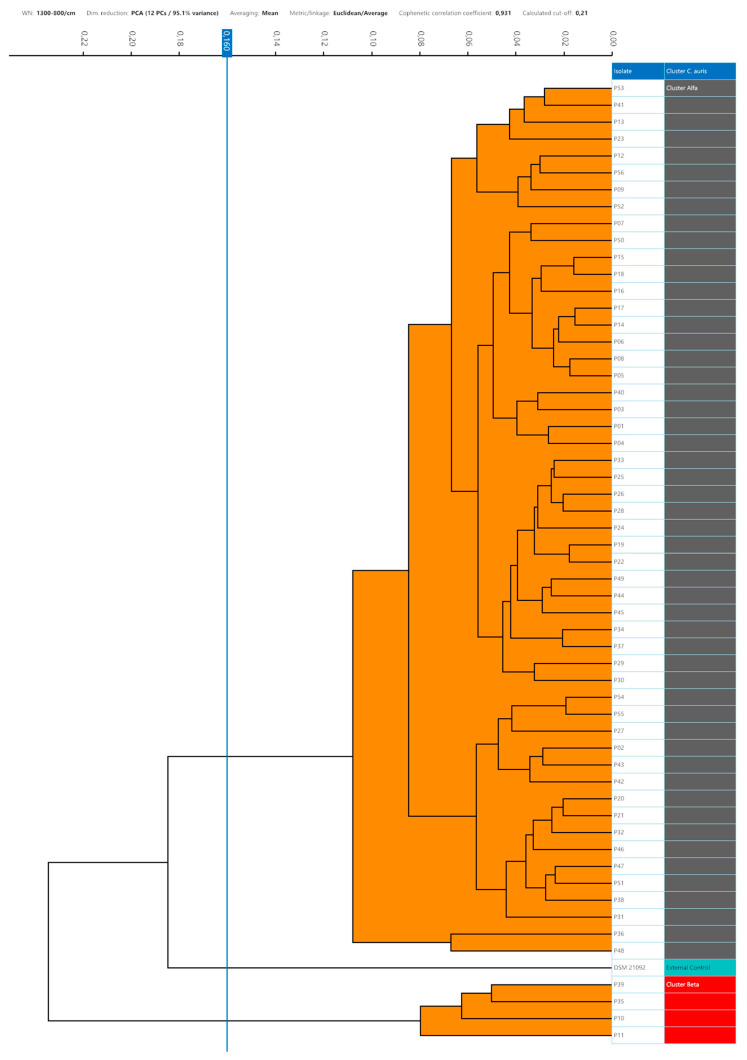
Dendrogram analysis of *C. auris* strains with a 0.160 cut-off setting. In the right column, the clusters are marked with different colours (grey, Alfa; red, Beta; green, external control).

Setting the 95th percentile value of 0.160 as the minimum clustering cut-off, two distinct clusters could be demonstrated by dendrogram and distance matrix analysis. The larger cluster, named Alfa, consisted of 52 (92.9%) strains strictly related to each other, and the smaller cluster, Beta, was formed by 4 (7.1%) similar strains. The third arm of the dendrogram was characterized by the external control spectra of *C. auris* interspersed between the Alfa and Beta clusters (Figure 3).

Considering the inter-isolate distances within the major Alfa cluster, the median and the 5th and 95th percentile values were 0.107, 0.061 and 0.180, respectively. The inter-isolate distance distributions among strains belonging to the same cluster consistently overlapped with each other and the intra-strain distribution (Figure 2). However, regarding the Beta cluster, because of the small number of isolates, the graphic distribution of inter-isolate distances was more irregular. Additional calculated variables and the intra-strain distributions are reported in the Supplementary Materials.

Through the inter-isolate distances analysis between the Beta and Alfa clusters, a Gaussian distribution of the distance values was observed with median and 5th and 95th percentile values of 0.244, 0.160, and 0.354, respectively. The inter-isolate distance analysis and graphic representation are described in detail in the Supplementary Materials. A summary of the principal calculated distance values is reported in Table 2.

**Figure 4:**
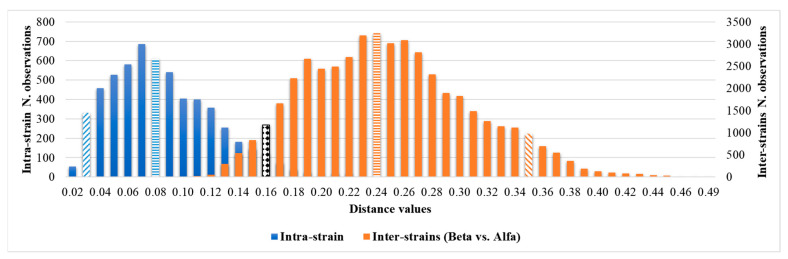
Comparison of intra-isolate and Beta vs. Alfa inter-isolate distances and clustering cut-off. The median is highlighted by horizontal rows, the 5th and 95th percentiles by oblique lines, and the overlapping minimum and maximum cut-offs, 0.160, are highlighted by the black and white texture.

The overlap between the 95th percentile of intra-isolates and the 5th percentile of the inter-cluster distribution can be observed in Table 2 and Figure 4.

No grey zone could be observed between the minimum and maximum clustering cut-off. Considering the external control position in the dendrogram representation, the cut-off values within which the external control could be considered independent of the Alfa cluster, or the Alfa and Beta clusters were <0.185 and <0.235, respectively. According to the 0.160 clustering cut-off, it was separated from both the Alfa and Beta clusters.

**Figure 5:**
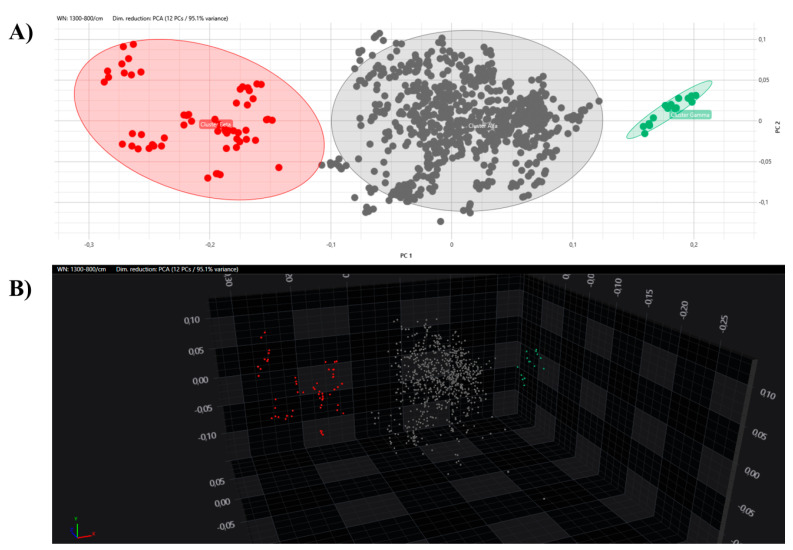
PCA analysis with 2D (**A**) and 3D (**B**) scatter plot representations. Clusters are marked with different colours (grey, Alfa; red, Beta; green, external control).

**Figure 6:**
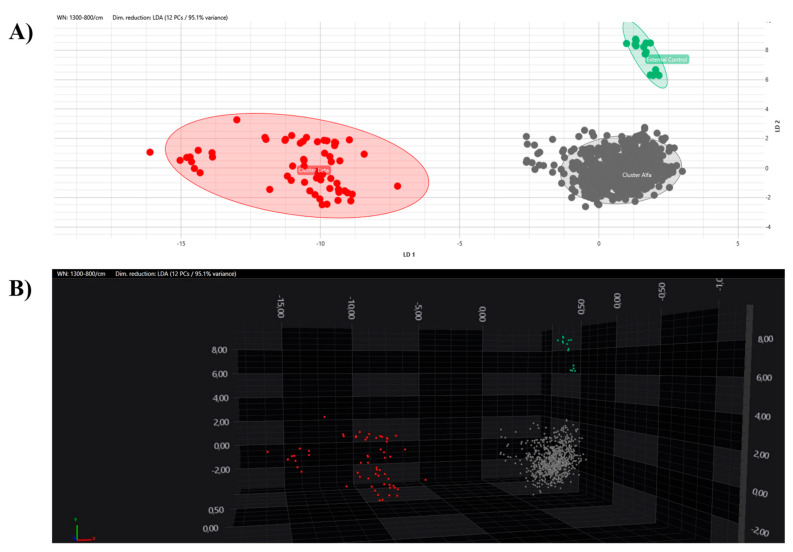
LDA analysis with 2D (**A**) and 3D (**B**) scatter plot representations. Clusters are marked with different colours (grey, Alfa; red, Beta; green, external control).

All the obtained spectra were successively analysed by PCA and LDA in 2D and 3D scatter plots. The acquired spectra are clearly grouped and separated from each other based on their respective clusters in both the PCA and LDA (Figure 5 and Figure 6).

A subset analysis was conducted to investigate strain features in relation to the samples, antifungal profiles, and time of isolation. No differences in the clustering analysis were observed among invasive and colonizing strains, antifungal profiles, or temporal series of the admitted patients and related samples.

## 4. Discussion

In recent years, *C. auris* has emerged as a multi-resistant fungal pathogen, with a significant clinical and health impact, and it has been recently reported as a critical issue on the WHO fungal priority list. The unique characteristic of this species is its ability to persist for a long time on human skin and in hospital environments, resulting in the spread of this microorganism inside and through health networks [22]. It is therefore of strategic importance to apply correct disinfection guidelines and protocols for the eradication and containment of this microorganism [23]. Also, its rapid and specific diagnosis, as well as the development of genotypical and phenotypic methods for typing, is pivotal in order to investigate the relationships among *C. auris* isolates involved in nosocomial outbreaks with accuracy and speed [24,25].

The main purpose of this study was the application of FT-IR spectroscopy to investigate the phylogenetic relationships among isolated strains from a local *C. auris* outbreak.

The first positive case of this emergent microorganism in the ICU ward led to the establishment of an extraordinary surveillance program aimed at identifying patients with *C. auris* colonization/infection. During the study period, an outbreak involving 56 patients was observed in the aforementioned ward. This phenomenon could be related to the high number of patients transferred from surrounding hospitals during the COVID-19 pandemic, as well as considerable access to transplant assessments and procedures of patients from other regions where *C. auris* had been previously found [11]. Furthermore, the high clinical severity at admission, the notable invasiveness of treatment (major surgery, extracorporeal support, renal replacement treatment), and the need for prolonged ICU length of stay and broad-spectrum antimicrobial therapies characterized the colonized population [11].

The innovative point of this study consisted of the definition of *C. auris* clustering cut-offs to discriminate between strains that belong to the same cluster and those that do not.

Assuming that a strain could be considered as part of the same cluster if its inter-strain distance is within the intra-strain distance, the distance between each replicate spectra, the 9^5^th percentile of intra-strain distance, was used to define the minimum clustering cut-off with a 5% of accepted classification error. The strains analysed by IR-BT, with the stated 0.160 cut-off, were found to belong to a maximum of two distinct clusters as follows: the major cluster, Alfa, consisting of 52 strains and comprising the index case, and the smaller cluster, Beta, consisting of the remaining 4 strains. Adopting this clustering cut-off, the external control was correctly positioned away from the Alfa and Beta clusters. Our minimum clustering cut-off is the result of a spectra evaluation of the 56 *C. auris* strains, and it is independent of local epidemiology and a clustering definition. For these reasons, the 0.160 cut-off value could be proposed as a robust and universal *C. auris* clustering cut-off, whether the incubation and analysis protocols are respected.

The comparison between the intra- and inter-isolate distances of the Alfa and Beta clusters shows a clear degree of overlapping distributions, supporting the common clonal origin of all the strains belonging to the clusters. The slight shift to the right of the inter-isolate distribution can be explained by the phenomenon of genetic drift due to the selective pressure to which *C. auris* is subjected in a hospital environment (e.g., use of antifungals, disinfectants, interactions with different hosts’ microbiota and immune systems), which is reflected in phenotypic changes in fungal macromolecule production.

Having ascertained the legitimacy of the two clusters, we focused on the maximum clustering cut-off definition. An inter-strains distance value analysis between the Beta and Alfa clusters was performed, assuming the fifth percentile of inter-isolate distance as the maximum clustering cut-off, with a 5% acceptance error. The inter-isolate distance distribution clearly shifted to the right in comparison with the intra-strain distribution, with only a small and partial overlap. The higher clustering cut-off was 0.160, coincident with the previous one, the minimum clustering cut-off. As a validation of the proposed clustering cut-off, the value within which the external control could be considered independent of the Alfa cluster or the Alfa and Beta clusters, in both the dendrogram and distance matrix analyses, was correctly identified over the defined 0.160 cut-off (<0.185). Moreover, both the PCA and LDA clearly confirmed the analysis and definition of the Alfa and Beta clusters. Interestingly, no difference in clustering analysis was observed considering colonizing or invasive strains, antimicrobial susceptibility profiles, specimen type, or sampling time. These results are confirmed by the similar clinical management of patients, both in terms of active administrated therapies, strain virulence, and patient outcomes.

The application of IR-BT as a typing tool for *Candida* spp. analysis was previously investigated in three studies. Contreras et al. in 2022, by applying a surveillance protocol to over 700 patients admitted to a third-level hospital in Los Angeles, identified 28 positive samples for *C. auris* within a year [26]. IR-BT typing allowed them to identify two clusters, the first consisting of 27 strains and the second consisting of one isolate [26]. The WGS analysis confirmed this subdivision and allowed them to classify the first cluster as belonging to the South African clade and the second as belonging to the South Asian clade [26]. In Vatanshenassan et al. study, 96 *C. auris* strains isolated from 14 geographical regions were analysed using five typing protocols (microsatellite analysis, sequencing of ITS regions, AFLP, MALDI-TOF, IR-BT) [2]. The results indicated microsatellite analysis as the preferred method for evaluating *C. auris* outbreaks [2]. The greater consistency with this method was the sequencing of ITS regions (45% similarity), followed by typing by IR-BT (33% similarity) [2]. The analysis of microsatellites allowed them to group the isolates into four clusters, corresponding to four geographical clades (East Asia, South Asia, South Africa and South America), while typing by IR-BT grouped the strains into two main clusters independently by their geographical origin [2].

The study performed by De Carolis et al. in 2023, and conducted at the University Hospital A. Gemelli IRCCS in Rome, analysed 59 strains of *C. parapsilosis* using IR-BT and microsatellite analysis [27]. The authors observed a concordance between the two methods varying from 47% to 74% in relation to the cut-off value used in IR-BT analysis (respectively, 0.967–0.995) [27].

The agreement discrepancies between microsatellite and IR-BT analysis observed in the two previous studies are presumably due to two reasons. The first lies in the analysis of the genetic regions responsible for fluconazole resistance: they contain different mutations depending on the *C. auris* clade, which can be demonstrated exclusively by genotypic analysis. The second reason is the different clonal origin of *C. auris* strains tested in the study by Vatanshenassan et al. [2]. In light of these characteristics, the greater concordance between genotypic (microsatellites) and phenotypic (IR-BT) analysis found by De Carolis et al. appears to be linked to the common geographical origin of the *C. parapsilosis* strains analysed, which are presumably genetically related [27]. This is also reflected in a lower rate of mutations responsible for resistance to fluconazole.

The common element of the three studies described above concerns the comparison between genetic analyses (WGS or microsatellite analysis) and IR-BT in order to evaluate their discriminatory capacity for clustering analysis. On the contrary, in our study, for the first time, a new analytical approach was developed to define a clustering cut-off for use in classifying new *C. auris* strains without the aid of genetic information. This could help to overcome the weaknesses of traditional typing techniques (WGS, pulsed-field gel electrophoresis, PGFE, MLST) such as access to these technologies, their costs and turn-around time, and the need for expert personnel, which are not commonly available in Microbiology laboratories. IR-BT could be a viable alternative for clustering analysis because it is cheaper, faster, reliable, and has a high daily throughput. In addition, its ability to use the cell surface as a sort of “barcode” that can provide more information than PGFE and MLST confirms the robustness of the method for industrial and clinical-epidemiological applications [25,28,29,30]. However, purchase and maintenance costs, as well as equipment size, make its implementation difficult in low-resource settings. Moreover, purification, cultivation, and sample preparation procedures are required, and validated cut-offs are not yet available [31].

## 5. Conclusions

In this study, IR-BT was used to investigate the onset and spread of an outbreak of *C. auris* at the University Hospital Città della Salute e della Scienza of Turin, Italy. The application of IR-BT based on FT-IR technology allowed us to obtain important information about the phylogenetic relationships among the analysed strains and to define, for the first time, a clustering cut-off with a “not WGS-based” statistical–mathematical approach, in order to classify *C. auris* strains as correctly belonging to the same cluster or not. The potential impact of this technology in the management of patients is pivotal not only for infection control policies but also for clinical application. The availability of information about *C. auris* circulating strains combined with their antifungal profiles and virulence, such as their colonizing vs. invasive behaviour, could bring important changes in patient therapeutic management.

Future perspectives will include confirmation of the results by WGS genotyping and the typing of new strains of *C. auris* in order to test and validate the robustness of the calculated cut-off. A high agreement between the two methods will allow for the use of IR-BT as a valid alternative to molecular methods because it is easy to use with a fast turnaround time, user-friendly interface, and low costs.

## Figures and Tables

**Table 1:** Patients with *C. auris* colonization and infection in relation to the different sample types.

Sample	Colonized Patients (%)	Infected Patients (%)
Skin swab	45 (80.4)	0 (0.0)
Urine	3 (5.4)	0 (0.0)
Tracheal aspirate	1 (1.8)	0 (0.0)
Sputum	1 (1.8)	0 (0.0)
Bronchoalveolar lavage	1 (1.8)	0 (0.0)
Blood	0 (0.0)	3 (5.4)
Intravascular catheter tip	0 (0.0)	2 (3.6)
Total	51 (91.1)	5 (8.9)

**Table 2:** Median and 5th and 95th percentile distance values in relation to the intra- or inter-strain analysis.

Distance Values	N. Strains	Median	5th Percentile	95th Percentile
Intra-strain	56	0.080	0.030	0.160
Inter-strains	56	0.115	0.062	0.266
Inter-strain cluster Alfa	52	0.107	0.061	0.180
Inter-strain cluster Beta	4	0.113	0.074	0.176
Inter-strain cluster Beta vs. cluster Alfa	56	0.244	0.160	0.354

## Data Availability

The original contributions presented in this study are included in this article/Supplementary Materials. Further inquiries can be directed to the corresponding author.

## Supplementary Materials

The following supporting information can be downloaded at https://www.mdpi.com/article/10.3390/microorganisms12071312/s1, Figure S1: Intra-strain distances values distribution; Figure S2: Distance matrix analysis of *C. auris* strains with 0.160 cut-off setting; Figure S3: Inter-strains distance values distribution of cluster Alfa strains; Figure S4: Inter-strains distance values distribution of cluster Beta strains; Figure S5: Inter-strains distances values distribution between cluster Beta and Alfa strains; Table S1: Intra-strain distances: mean, minimum, maximum, standard deviation, median, 5th and 95th percentile for each isolate; Table S2: Inter-strains distance values of the cluster Alfa: mean, minimum, maximum, standard deviation, median, 5th and 95th percentile for each isolate; Table S3: Inter-strains distance values of the cluster Beta: mean, minimum, maximum, standard deviation, median, 5th and 95th percentile for each isolate; Table S4: Inter-strains distance values of cluster Beta versus cluster Alfa: mean, minimum, maximum, standard deviation, median, 5th and 95th percentile.
