# Supplementary material for: Fourier Transform Infrared Spectroscopy Application for Candida auris Outbreak Typing in a Referral Intensive Care Unit: Phylogenetic Analysis and Clustering Cut-Off Definition
Source: Microorganisms. 2024 Jun 27;12(7):1312. doi: 10.3390/microorganisms12071312 (PMC11279149; doi:10.3390/microorganisms12071312)
Supplement: Supplementary file 1 [file microorganisms-12-01312-s001.zip › microorganisms-3056136-supplementary.pdf]

SUPPLEMENTARY MATERIAL

Intra-strain analysis results

For each strain of *C. auris* under study, intra-isolate distance values of mean, minimum, maximum, standard

deviation, median, 5th and 95th percentile, were calculated and reported in Table S1.

Table S1. Intra-strain distances: mean, minimum, maximum, standard deviation, median, 5th and 95th percentile for each isolate

Strain	Average	Minimum	Maximum	Standard deviation	Median	5th percentile	95th percentile
P01	0.082	0.030	0.170	0.032	0.090	0.040	0.130
P02	0.065	0.020	0.110	0.021	0.060	0.030	0.101
P03	0.075	0.020	0.140	0.026	0.070	0.032	0.120
P04	0.076	0.020	0.120	0.025	0.080	0.030	0.110
P05	0.077	0.020	0.120	0.030	0.080	0.030	0.120
P06	0.084	0.020	0.130	0.029	0.090	0.030	0.130
P07	0.113	0.030	0.260	0.053	0.100	0.040	0.228
P08	0.088	0.030	0.140	0.034	0.090	0.040	0.130
P09	0.064	0.030	0.090	0.016	0.070	0.040	0.090
P10	0.064	0.030	0.100	0.017	0.060	0.040	0.090
P11	0.110	0.030	0.180	0.049	0.090	0.042	0.170
P12	0.063	0.030	0.090	0.017	0.060	0.040	0.090
P13	0.108	0.030	0.160	0.041	0.120	0.032	0.158
P14	0.071	0.030	0.140	0.022	0.070	0.040	0.110
P15	0.087	0.020	0.140	0.032	0.080	0.030	0.138
P16	0.127	0.030	0.240	0.049	0.130	0.042	0.220
P17	0.081	0.020	0.150	0.032	0.080	0.030	0.130
P18	0.090	0.030	0.150	0.032	0.100	0.030	0.130
P19	0.098	0.030	0.150	0.041	0.100	0.030	0.150
P20	0.071	0.030	0.110	0.024	0.070	0.030	0.110
P21	0.079	0.020	0.110	0.024	0.090	0.040	0.110
P22	0.110	0.030	0.180	0.044	0.120	0.032	0.160
P23	0.065	0.030	0.120	0.019	0.070	0.030	0.090
P24	0.098	0.020	0.150	0.038	0.110	0.030	0.140
P25	0.111	0.020	0.180	0.042	0.120	0.030	0.160

P26	0.119	0.020	0.180	0.053	0.130	0.030	0.180
P27	0.078	0.030	0.120	0.025	0.080	0.040	0.110
P28	0.124	0.030	0.210	0.052	0.140	0.030	0.190
P29	0.103	0.020	0.180	0.039	0.110	0.040	0.160
P30	0.099	0.030	0.170	0.039	0.110	0.030	0.160
P31	0.116	0.020	0.220	0.061	0.100	0.030	0.200
P32	0.096	0.020	0.160	0.041	0.100	0.030	0.150
P33	0.132	0.020	0.220	0.063	0.130	0.030	0.220
P34	0.077	0.020	0.130	0.033	0.070	0.030	0.120
P35	0.086	0.030	0.140	0.032	0.080	0.040	0.130
P36	0.079	0.030	0.130	0.026	0.080	0.030	0.120
P37	0.062	0.030	0.100	0.016	0.060	0.030	0.090
P38	0.100	0.020	0.210	0.047	0.120	0.030	0.168
P39	0.130	0.030	0.360	0.085	0.120	0.040	0.338
P40	0.061	0.020	0.100	0.014	0.060	0.040	0.080
P41	0.061	0.020	0.100	0.019	0.060	0.030	0.090
P42	0.093	0.030	0.300	0.067	0.070	0.040	0.258
P43	0.093	0.020	0.190	0.042	0.090	0.030	0.178
P44	0.076	0.020	0.120	0.025	0.080	0.030	0.108
P45	0.064	0.020	0.100	0.019	0.070	0.030	0.090
P46	0.090	0.020	0.200	0.040	0.085	0.030	0.170
P47	0.076	0.020	0.120	0.022	0.080	0.032	0.110
P48	0.094	0.020	0.170	0.041	0.090	0.030	0.150
P49	0.057	0.030	0.090	0.012	0.060	0.040	0.070
P50	0.070	0.020	0.130	0.022	0.070	0.040	0.110
P51	0.066	0.020	0.120	0.022	0.070	0.030	0.100
P52	0.080	0.020	0.120	0.024	0.090	0.040	0.110
P53	0.082	0.030	0.130	0.033	0.070	0.040	0.120
P54	0.082	0.020	0.140	0.025	0.090	0.030	0.110
P55	0.066	0.020	0.120	0.021	0.070	0.022	0.090
P56	0.086	0.020	0.150	0.029	0.080	0.040	0.130
**Total**	**0.087**	**0.020**	**0.360**	**0.041**	**0.080**	**0.030**	**0.160**

The histogram of the frequencies of intra-strain values with unimodal trend is reported in Figure S1.

Figure S1**.** Intra-strain distances values distribution

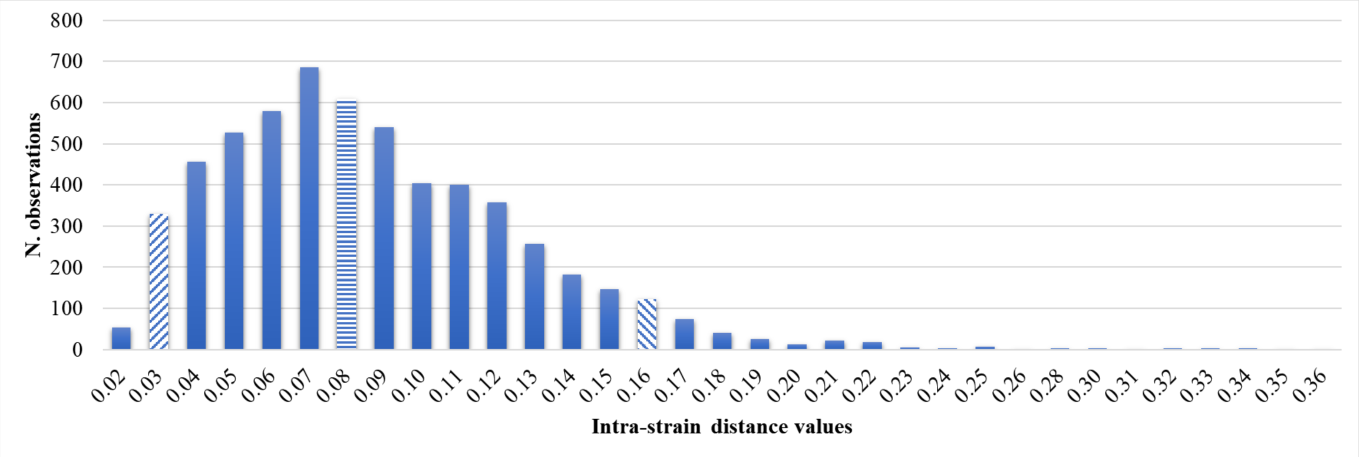

Median is highlighted by horizontal rows, 5th and 95th percentile by oblique lines

Distance matrix representation of analysed spectra is reported in Figure S2. The shades of colour from dark red to white to dark blue represent an increase in the distances between the measured spectra. Lowest values, represented in dark red, are close to zero. The darkest edges define the 3 clusters.

Figure S2. Distance matrix analysis of *C. auris* strains with 0.160 cut-off setting

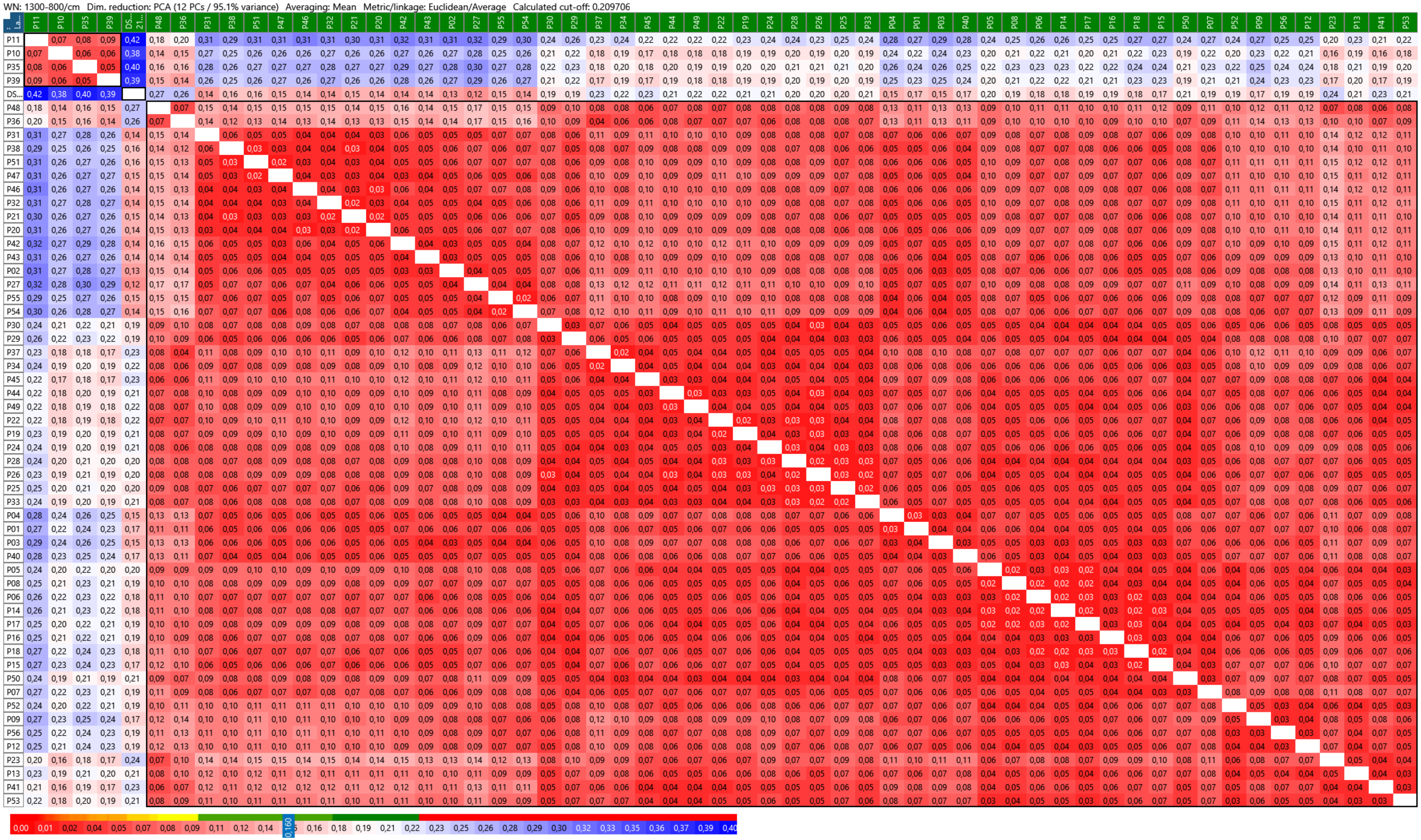

Inter-isolates analysis results

Cluster Alfa

Inter-isolates distance mean, minimum and maximum, standard deviation, median, 5th and 95th percentile values of the 52 *C. auris* strains belonging to the cluster Alfa are shown in Table S2.

Table S2. Inter-strains distance values of the cluster Alfa: mean, minimum, maximum, standard deviation, median, 5th and 95th percentile for each isolate

Strain	Average	Minimum	Maximum	Standard deviation	Median	5th percentile	95th percentile
P01	0.104	0.036	0.287	0.031	0.099	0.062	0.160
P02	0.107	0.024	0.263	0.035	0.103	0.057	0.172
P03	0.102	0.028	0.295	0.033	0.097	0.057	0.163
P04	0.105	0.034	0.316	0.032	0.101	0.059	0.163
P05	0.103	0.029	0.288	0.032	0.099	0.055	0.159
P06	0.101	0.030	0.295	0.031	0.098	0.057	0.157
P07	0.118	0.035	0.317	0.047	0.109	0.058	0.233
P08	0.106	0.029	0.300	0.034	0.102	0.058	0.169
P09	0.110	0.037	0.287	0.033	0.104	0.067	0.177
P12	0.105	0.031	0.304	0.032	0.100	0.062	0.167
P13	0.121	0.030	0.330	0.043	0.111	0.065	0.202
P14	0.097	0.030	0.288	0.029	0.092	0.057	0.149
P15	0.104	0.030	0.281	0.033	0.100	0.058	0.163
P16	0.121	0.032	0.315	0.041	0.116	0.064	0.201
P17	0.102	0.028	0.300	0.031	0.098	0.057	0.157
P18	0.104	0.035	0.281	0.032	0.101	0.057	0.164
P19	0.115	0.026	0.312	0.035	0.110	0.068	0.177
P20	0.112	0.030	0.283	0.035	0.107	0.062	0.176
P21	0.111	0.024	0.288	0.035	0.106	0.061	0.173
P22	0.121	0.028	0.308	0.038	0.117	0.066	0.185
P23	0.124	0.035	0.317	0.036	0.125	0.067	0.182
P24	0.113	0.024	0.279	0.035	0.111	0.059	0.171
P25	0.115	0.026	0.297	0.037	0.111	0.062	0.178
P26	0.120	0.024	0.308	0.036	0.118	0.062	0.180
P27	0.119	0.027	0.288	0.040	0.114	0.063	0.191
P28	0.121	0.024	0.317	0.039	0.121	0.059	0.184

P29	0.111	0.023	0.276	0.035	0.107	0.061	0.173
P30	0.111	0.030	0.320	0.031	0.107	0.064	0.168
P31	0.133	0.024	0.297	0.043	0.133	0.064	0.204
P32	0.112	0.024	0.317	0.037	0.107	0.059	0.176
P33	0.127	0.023	0.309	0.044	0.120	0.065	0.203
P34	0.105	0.030	0.276	0.037	0.098	0.055	0.175
P36	0.134	0.038	0.283	0.039	0.133	0.071	0.194
P37	0.110	0.033	0.293	0.031	0.109	0.062	0.161
P38	0.120	0.026	0.322	0.040	0.118	0.059	0.187
P40	0.095	0.032	0.288	0.029	0.090	0.057	0.151
P41	0.108	0.030	0.289	0.032	0.107	0.059	0.163
P42	0.123	0.029	0.331	0.052	0.114	0.059	0.247
P43	0.116	0.036	0.273	0.039	0.111	0.061	0.187
P44	0.105	0.033	0.305	0.028	0.103	0.064	0.151
P45	0.106	0.030	0.285	0.031	0.103	0.061	0.161
P46	0.113	0.023	0.330	0.038	0.108	0.060	0.179
P47	0.114	0.022	0.284	0.037	0.108	0.061	0.181
P48	0.143	0.046	0.331	0.040	0.143	0.081	0.207
P49	0.100	0.031	0.290	0.027	0.099	0.059	0.145
P50	0.100	0.030	0.283	0.029	0.098	0.057	0.151
P51	0.111	0.022	0.281	0.038	0.106	0.058	0.180
P52	0.111	0.035	0.312	0.033	0.107	0.065	0.173
P53	0.109	0.031	0.311	0.038	0.102	0.059	0.183
P54	0.116	0.026	0.315	0.037	0.111	0.066	0.186
P55	0.107	0.026	0.302	0.035	0.102	0.060	0.175
P56	0.115	0.035	0.317	0.036	0.110	0.065	0.183
**Total**	**0.113**	**0.022**	**0.331**	**0.037**	**0.107**	**0.061**	**0.180**

Inter-isolate distance values of the larger cluster Alfa are graphically reported in the histogram of the frequencies in Figure S3. A normal distribution was observed.

Figure S3. Inter-strains distance values distribution of cluster Alfa strains

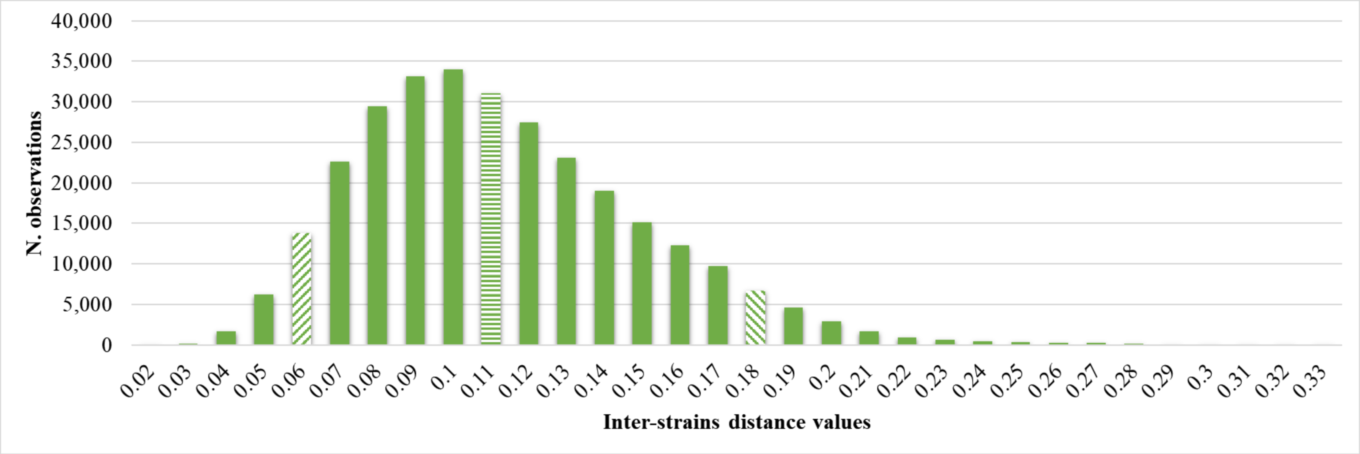

Median is highlighted by horizontal rows, 5th and 95th percentile by oblique lines

Cluster Beta

Inter-isolates distance mean, minimum and maximum, standard deviation, median, 5th and 95th percentile values of the 4 *C. auris* strains belonging to the cluster Beta are shown in Table S3.

Table S3. Inter-strains distance values of the cluster Beta: mean, minimum, maximum, standard deviation, median, 5th and 95th percentile for each isolate

Strain	Average	Minimum	Maximum	Standard deviation	Median	5th percentile	95th percentile
P10	0.112	0.054	0.380	0.042	0.104	0.073	0.147
P11	0.132	0.061	0.388	0.044	0.131	0.077	0.180
P35	0.115	0.043	0.374	0.044	0.105	0.073	0.162
P39	0.128	0.043	0.388	0.066	0.110	0.073	0.337
**Total**	**0.121**	**0.043**	**0.388**	**0.051**	**0.113**	**0.074**	**0.176**

Inter-isolate distance values of the minor cluster Beta are graphically reported in the histogram of the frequencies in Figure S4. The distribution wasn't normal due to the small number of strains involved in the cluster.

Figure S4. Inter-strains distance values distribution of cluster Beta strains

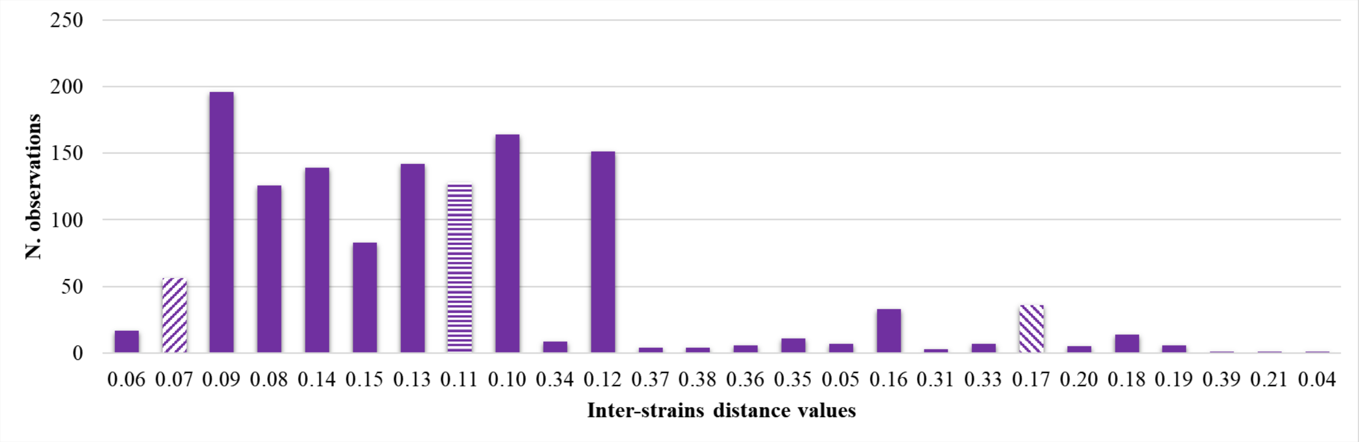

Median is highlighted by horizontal rows, 5th and 95th percentile by oblique lines

Cluster Beta versus cluster Alfa

Inter-isolates distance mean, minimum and maximum, standard deviation, median, 5th and 95th percentile values between cluster Beta and Alfa strains are shown in Table S4.

Table S4. Inter-strains distance values of cluster Beta versus cluster Alfa: mean, minimum, maximum, standard deviation, median, 5th and 95th percentile

Strain	Average	Minimum	Maximum	Standard deviation	Median	5th percentile	95th percentile
P10	0.229	0.119	0.409	0.044	0.228	0.156	0.301
P11	0.275	0.105	0.446	0.065	0.282	0.162	0.370
P35	0.245	0.120	0.417	0.052	0.243	0.169	0.341
P39	0.247	0.091	0.491	0.063	0.241	0.156	0.373
**Total**	**0.249**	**0.091**	**0.491**	**0.059**	**0.244**	**0.160**	**0.354**

Inter-isolates distance values of cluster Beta *versus* Alfa are graphically reported in the histogram of the frequencies in Figure S5. A normal distribution was observed.

Figure S5. Inter-strains distances values distribution between cluster Beta and Alfa strains

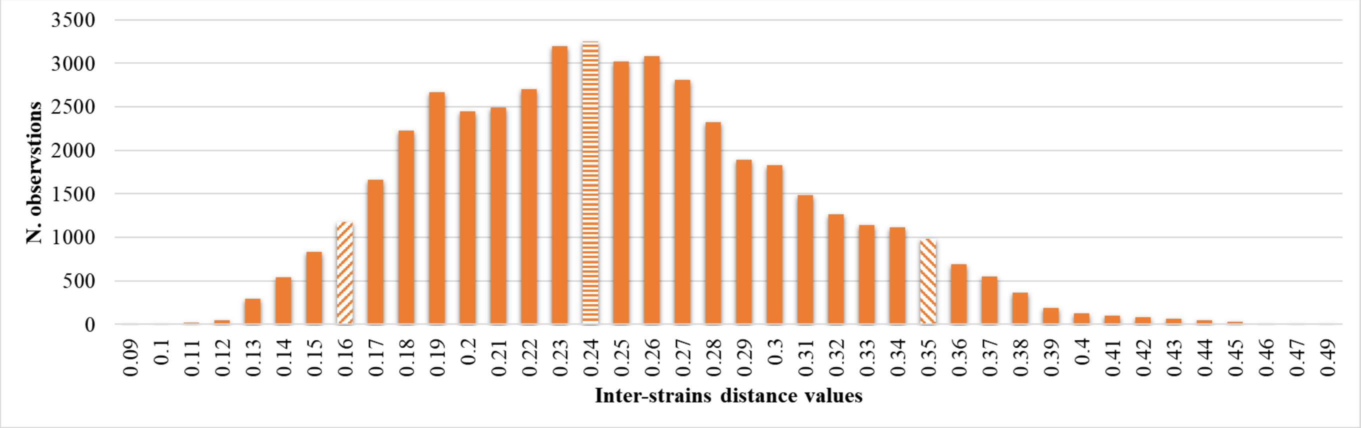

Median is highlighted by horizontal rows, 5th and 95th percentile by oblique lines
